# The Use of Complementary and Alternative Medicine by Korean Breast Cancer Women: Is It Associated with Severity of Symptoms?

**DOI:** 10.1155/2015/182475

**Published:** 2015-12-03

**Authors:** Jung Hye Hwang, Woon-Yong Kim, Mansoor Ahmed, Soojeung Choi, Jiwoo Kim, Dong Woon Han

**Affiliations:** ^1^Department of Obstetrics and Gynecology, Hanyang University, College of Medicine, 222 Wangsimni-ro, Seongdong-gu, Seoul 04763, Republic of Korea; ^2^Institute of Health Services Management, Hanyang University, 222 Wangsimni-ro, Seongdong-gu, Seoul 04763, Republic of Korea; ^3^Department of Global Health and Department of Preventive Medicine, Hanyang University, College of Medicine, 222 Wangsimni-ro, Seongdong-gu, Seoul 04763, Republic of Korea

## Abstract

*Background*. Use of complementary and alternative medicine (CAM) among patients with breast cancer could be associated with severity of the cancer symptoms experienced, but there is little evidence to prove this. This study tried to investigate any difference in the severity of breast cancer symptoms between CAM users and nonusers.* Methods*. The study followed cross-sectional design using structured survey questionnaire. Survey participants were recruited from four different healthcare settings in Seoul, South Korea. The survey instrument comprised 39 items including questions on demographics, use of CAM, and six main symptoms associated with breast cancer and cancer treatment.* Results*. Out of 288 participants, 67% stated using one or more modalities of CAM. Age, education, and time duration since diagnosis of cancer were significantly associated with use of CAM. About 90% of the CAM users experienced side effects of cancer treatment. CAM users reported more severe anxiety and skin/hair changes than nonusers.* Conclusions*. CAM was used by those breast cancer patients who experience more severe symptoms to alleviate the conditions associated with breast cancer and cancer treatment. Our findings revealed motivation behind the CAM use, which has profound implications for clinicians to recognize patient-perceived needs.

## 1. Introduction

Breast cancer is a chronic disease associated with severe physical and emotional burden [1]. Results of a five-year observational cohort study in the UK showed presence of depression, anxiety, or both in nearly 50% of the women with breast cancer [2], and pain, fatigue, anorexia, and skin changes are common over the course of the disease and its treatment [3]. For managing these cancer-associated symptoms, there are few approved standard guidelines; therefore, as compared to patients with other cancers, use of complementary and alternative medicine (CAM) is most common among breast cancer women [4]. Such increase in popularity of CAM might be due to the breast cancer patients' dissatisfaction with conventional therapies so they could integrate it with use of CAM [5]. Furthermore, research suggests that symptom management is one of the most common reasons behind CAM use among women with breast cancer [6, 7]. We hypothesize that use of CAM could be associated with severity of symptoms experienced by women with breast cancer. However, no conclusive research has been conducted to establish relationship between use of CAM and severity of symptoms associated with breast cancer and its treatment [8].

A number of studies have underscored high prevalence of CAM among women with breast cancer [9–12]. The variety of CAM therapies used and the factors related to CAM use have also been recognized by several researchers. A study reported that dietary supplements are the most preferred type of CAM used by women with breast cancer, while another study reported that meditation, herbs, and spiritual healing are the most commonly used modalities of CAM among such patients [13, 14]. Wanchai et al. demonstrated that women with breast cancer with higher level of education, higher income level, and younger age have increased tendency to be consumers of CAM [15].

In Korea, breast cancer is the most common type of cancer among female population and the prevalence has been increasing continuously [16]. Moreover, the most recent report reveals that more than 50% of the Korean women with breast cancer experience cancer-associated anxiety and depression [17]. Few studies have focused on the use of CAM among women with breast cancer in Korea; however, they did not investigate why these women use CAM. A survey on 160 Korean women with breast cancer highlighted the CAM usage by 36% of the participants [18]. The more recent survey conducted on the breast cancer patients in Korea reported the use of CAM by 57.4% of the patients [19]. The study discussed only quality of life indicators among CAM users and nonusers [19]. To the best of our knowledge, the surveys conducted in Asian populations so far have not studied differences in the severity of symptoms associated with breast cancer and its treatment between users of CAM and nonusers. The aim of this study is to evaluate the use of CAM in the Korean women with breast cancer by investigating differences in the severity of self-reported symptoms associated with breast cancer and its treatment between users of CAM and nonusers. Additionally, the study explored CAM users' attitudes towards CAM and conventional healthcare.

## 2. Methods

### 2.1. Participants

The study followed cross-sectional design to investigate the CAM use among South Korean female breast cancer survivors. Structured questionnaire was used to conduct face-to-face survey for three weeks from May 31, 2012, to June 20, 2012. The subjects were Korean female breast cancer patients aged over 18 years and were invited to participate in the study when they visited outpatient department of four hospitals located in Seoul, namely, Hanyang University Seoul Hospital, Hanyang University Guri Hospital, Cheil General Hospital and Women's Healthcare Centre, and Jeongpajong Breast and Thyroid Clinic. A total of 300 subjects were invited and all of them agreed to participate in the survey. Given that 12 participants did not complete the questionnaire, data of only 288 participants were included in the final analysis.

Ethical approval for the study was obtained from the Institutional Review Board on Human Subjects Research and Ethics Committees, Hanyang University Guri Hospital. Prior to the survey, all participants provided IRB-approved written informed consent.

### 2.2. Data Collection

In order to conduct the survey in each of the facilities, four surveyors were trained. The survey questionnaire, developed in the Korean language, is comprised of a total of 39 items. Questions were asked on use of CAM, demographic characteristics, CAM users' attitudes towards conventional health care, sources of information, and severity of six self-reported symptoms, namely, pain, fatigue, depression, anorexia, skin and hair changes, and anxiety. Severity of each symptom was rated on a 6-point Likert scale from 0 to 5 (no symptom to most severe). All of the participants were asked to respond to questions on demographic characteristics, CAM use, and severity of symptoms, whereas questions related to attitudes, types of CAM, source of information, and communication were asked from CAM users only.

### 2.3. Statistical Analysis

Descriptive statistics (using number and percentage) were used to describe severity of symptoms, CAM users' attitudes, types of CAM, and sources of information. Linear logistic regression analysis was created (assuming confidence interval of 95%) to see any association between participants' characteristics and use of CAM. In the regression model, we considered use of CAM as dependent variable whereas independent variables comprising age, religion, education, marital status, employment, household income, family history of cancer, diagnostic institution, method of diagnosis, and time since breast cancer were diagnosed. We selected significance level of *α* = 0.05 for all analyses. The data were analyzed using Statistical Package for Social Sciences (SPSS) v. 18.0.

## 3. Results

### 3.1. Demographics

Table 1 displays characteristics of 288 study subjects. In terms of characteristics, 37.2% were aged >54 years, 58.7% followed Western religions (Roman Catholic, Christian), 62.5% had education up to high school, 82.6% were married, 55.2% were unemployed, 63.2% had household income <5 million KRW, 72.9% had no family history of cancer, and 54.2% were residents of Seoul.

### 3.2. Utilization of CAM and Predictors

In total, 193 (67%) participants stated utilizing one or more modalities of CAM (Table 1). Participants who were more likely to use CAM were in the age between 46 and 54 years (39.4%) and were followers of Western religions (63.7%), of higher school or lower education level (58.5%), married (84.5%), unemployed (51.3%), with household income <5 million won/month (57.5%), without family history of cancer (72%), residents of Seoul (59.1%), and with cancer diagnosed at facilities other than tertiary care hospitals (61.1%), cancer detected by screening (56.5%), and cancer diagnosed since 12–36 months (33.7%). Logistic regression analysis revealed that age, level of education, and time since diagnosis were significantly associated with use of CAM.

### 3.3. Severity of Symptoms

Table 2 presents Likert scale data with full frequency distributions of the scores on severity of self-reported symptoms among CAM users and nonusers. The proportion of high severity of symptoms is relatively higher among CAM users than among the nonusers, especially in the case of anxiety and skin/hair changes. Similarly, proportion of participants without symptoms is relatively higher among nonusers than among CAM users for all symptoms.

### 3.4. Attitudes towards CAM

Table 3 shows information on CAM users' attitudes and practices regarding CAM. Out of 193 CAM users, 39.4% believed that CAM prevents progression of breast cancer and promotes health and 32.1% used it to mitigate symptoms and 19.2% used it to enhance the immune system. Moreover, 65.3% did not believe that CAM reduces costs for the treatment of breast cancer and only 32.1% believed that CAM reduces overall health care utilization. When asked who recommended them to use CAM, 40.4% responded that it was their own decision, 30.6% were advised by their family, and only 8.8% were suggested by their respective healthcare provider whereas only 48.7% believed that CAM helps in the treatment of breast cancer and 62.7% had plans to continue using any of the CAM therapies.

### 3.5. Attitudes towards Conventional Health Care

CAM users' attitudes towards conventional health care are illustrated in Table 4. Out of 193 CAM users, 61.7% did not disclose to their healthcare providers. The reasons for not doing so were given as follows: there is no need to discuss (63.9%) and healthcare provider may dislike it (30.3%). Eighty-nine percent of the CAM users in our study had experienced one or more side effects of the conventional therapy for breast cancer.

### 3.6. Modalities of CAM Use

Modalities of CAM used by study participants are shown in Table 5. The most common types of CAM were exercise and yoga (53.9%), ginseng (53.4%), mushrooms (46.6%), beans (43.5%), multivitamins (40.4%), and prayer, meditation, and Zen (34.2%). In aggregate, dietary and nutritional therapies were most popularly consumed, followed by mind-body interventions, alternative medicine, and biological supplements. Massage was most popularly used (20.2%) among manipulative and body-based therapies. While among alternative medicine therapies, Traditional Korean Herbal Medicine (23.3%) and acupuncture (22.3%) were most commonly used by the study participants.

## 4. Discussion

This study reports the recent trend of CAM use among breast cancer survivors in South Korea. Results of our study show high prevalence of CAM utilization by 67% of the participants, which is consistent with previous studies [15, 20, 21]. However, this rate is slightly lower when compared to use of CAM among general population in Korea [22]. Consistent with previous studies, likely predictors of CAM use included age, level of education, and time since diagnosis of breast cancer [10, 15, 23].

Patterns of CAM modalities used among the study participants reflect their sociocultural background as ginseng was the most popular among nutritional therapies (53.4%), whereas the previous Korean study reported its consumption as 33.6% [19]. Overall, dietary and nutritional therapies were the most commonly consumed category of CAM, consistent with the study conducted by Fasching et al. [24]. We also found that 38.3% of the CAM users, at some point, discussed CAM use with their healthcare provider. Previous Korean studies reported this number at 20% and 29.6%, respectively [18, 19]. These numbers suggest increase in disclosure of CAM use to healthcare providers in the country.

Our results show that 90% of the CAM users experienced side effects of conventional therapy for cancer, which is supported by previous studies [25, 26]. The breast cancer patients used CAM to complement conventional medical treatment, which is further confirmed by our results where majority of the CAM users believed that using CAM reduces neither overall costs of care nor healthcare utilization. Thus, as our results illustrate, participants used CAM mainly to prevent progress of disease and promote health, which is similar to findings from previous studies conducted in Korea and Malaysia [19, 27]. Another major reason for using CAM was to alleviate symptoms and to enhance immune systems, which is similar to previous research [10, 28]. In order to see any difference between CAM users and nonusers, we compared the two groups with relation to severity of self-reported symptoms.

Based on the literature review and our clinical experience [2, 3, 17], we selected six symptoms for investigation: pain, fatigue, depression, anorexia, anxiety, and skin and hair changes. These symptoms can seriously affect breast cancer patients' quality of life, which they experience over the course of disease and its treatment [29, 30]. Our study found significant difference in the severity of fatigue, depression, anxiety, and skin and hair changes between CAM users and nonusers. Our results imply that CAM is used by those breast cancer patients who experience more severe symptoms. Moreover, these findings are in agreement with other results of this study where symptom management is one of the major reasons for CAM use. Previous studies also show that breast cancer patients use CAM to alleviate their symptoms [28, 31]. A study conducted in the UK found that breast cancer patients undergoing radiotherapy believed that CAM would mitigate their fatigue and skin changes [32]. About 54% of CAM users in our study were using yoga as complementary therapy. There is strong evidence of yoga therapy improving fatigue in breast cancer survivors [33]. Moreover, yoga therapy can also mitigate anxiety and other symptoms among breast cancer outpatients undergoing conventional radiotherapy and chemotherapy [34] whereas a study conducted in the United States established relationship between restorative yoga and self-reported emotional symptoms and fatigue among breast cancer patients [35]. More than 53% of the CAM users in our sample used ginseng, which is commonly used for symptoms associated with breast cancer in Asian countries [36].

Our study has produced important findings regarding the motivation behind use of CAM among breast cancer patients. Patients, who experienced more severe symptoms, might have wanted to alleviate them via using CAM, whereas patients with milder symptoms did not use CAM. Results of the study have significant implications for clinical oncology as there is evidence of side effects from CAM use [37]. Furthermore, results of the study may enable oncologists to identify breast cancer patients' needs and expectations which are otherwise difficult in the absence of strong evidence supporting the effectiveness of CAM use in such patients [38]. We recommend that oncologists should be aware of the extent to which breast cancer patients use CAM and reasons for its usage. Moreover, oncologists should initiate the communication with their patients especially those who experience more severe symptoms as our results show that a large number of patients do not disclose their use of CAM. Findings of our study have given more insight into the trend of using CAM among breast cancer patients. Further studies are suggested using larger samples in different settings to more investigate the relationship between CAM use and symptoms associated with breast cancer and its treatment.

## 5. Conclusions

CAM is used by breast cancer patients who experience more severe symptoms associated with breast cancer and its treatment to alleviate the symptoms. Patients' perceptions and understanding of their illness, and health benefits from CAM use, are an evolving process that may have an influence on quality of life and health behavior choices. It remains to be discovered whether this motivation behind the use of CAM is in line with true medical benefit. As new information about CAM becomes available, it will be important to develop and provide education programs for health professionals and to increase awareness among patients.

## Figures and Tables

**Table 1:** Participants' characteristics according to CAM use.

Characteristics	CAM users		Nonusers		Total		*P* value
	*N*	%	*N*	%	*N*	%	
	193	67.0	95	33.0	288	100.0	
Age group							0.003
<46	59	30.6	27	28.4	86	29.9	
46–54	76	39.4	19	20.0	95	33.0	
>54	58	30.1	49	51.6	107	37.2	
Religion							0.122
Western	123	63.7	46	48.4	169	58.7	
Eastern	70	36.3	49	51.6	119	41.3	
Education							0.027
≤High school	113	58.5	67	70.5	180	62.5	
≥University	80	41.5	28	29.5	108	37.5	
Marital status							0.455
Single	30	15.5	20	21.1	50	17.4	
Married	163	84.5	75	78.9	238	82.6	
Employed							0.320
No	99	51.3	60	63.2	159	55.2	
Yes	94	48.7	35	36.8	129	44.8	
Household income							0.797
<5 million won/month	111	57.5	71	74.7	182	63.2	
≥5 million won/month	82	42.5	24	25.3	106	36.8	
Family history of cancer							0.465
No	139	72.0	71	74.7	210	72.9	
Yes	54	28.0	24	25.3	78	27.1	
Residence							0.537
Seoul	114	59.1	42	44.2	156	54.2	
Elsewhere	79	40.9	53	55.8	132	45.8	
Where diagnosed							0.119
Tertiary care hospitals	75	38.9	45	47.4	120	41.7	
Other	118	61.1	50	52.6	168	58.3	
Detected by							0.616
Screening	109	56.5	65	68.4	174	60.4	
Symptoms	84	43.5	30	31.6	114	39.6	
Months since diagnosis							0.036
<12	47	24.4	32	33.7	79	27.4	
12–36	65	33.7	26	27.4	91	31.6	
37–60	38	19.7	18	18.9	56	19.4	
>60	43	22.3	19	20.0	62	21.5	

^*∗*^5 million Korean won (KRW) was about 4,500 US dollars at the time of survey.

**Table 2:** Severity of self-reported symptoms between CAM users (193) and nonusers (95): *N* (%).

Score	Pain		Fatigue		Depression		Anorexia		Skin/hair changes		Anxiety	
	CAM user	Nonuser	CAM	Non	CAM	Non	CAM	Non	CAM	Non	CAM	Non
0	47 (24.4)	24 (25.3)	19 (9.8)	17 (17.9)	28 (14.5)	21 (22.1)	22 (11.4)	13 (13.7)	26 (13.5)	20 (21.1)	21 (10.9)	13 (13.7)
1	52 (26.9)	31 (32.6)	49 (25.4)	35 (36.9)	47 (24.4)	32 (33.7)	41 (21.2)	28 (29.5)	46 (23.8)	36 (37.9)	42 (21.8)	31 (32.6)
2	17 (8.8)	8 (8.4)	13 (6.7)	1 (1.1)	11 (5.7)	3 (3.2)	13 (6.7)	2 (2.1)	15 (7.8)	3 (3.2)	17 (8.8)	8 (8.4)
3	33 (17.1)	12 (12.6)	49 (25.4)	19 (20)	50 (25.9)	17 (17.9)	40 (20.7)	20 (21.1)	30 (15.5)	15 (15.8)	35 (18.1)	21 (22.1)
4	19 (9.8)	11 (11.6)	35 (18.1)	11 (11.6)	30 (15.5)	11 (11.6)	38 (19.7)	15 (15.8)	33 (17.1)	9 (9.5)	41 (21.2)	10 (10.5)
5	25 (13.0)	9 (9.5)	28 (14.5)	12 (12.6)	27 (14)	11 (11.6)	39 (20.2)	17 (17.9)	43 (22.3)	12 (12.6)	37 (19.2)	12 (12.6)

**Table 3:** CAM users' attitudes towards CAM.

Category	*N*	%
Reason for using CAM		
For cancer treatment	14	7.3
To prevent progress of disease	76	39.4
To mitigate symptoms	62	32.1
To enhance immune system	37	19.2
Other	4	2.1
Reduces overall costs		
No	126	65.3
Yes	67	34.7
Reduces healthcare utilization		
No	131	67.9
Yes	62	32.1
CAM recommended by		
Myself	78	40.4
Family	59	30.6
Breast cancer patients	20	10.4
Other relatives or friends	17	8.8
My healthcare provider	15	7.7
Other	4	2.1
CAM helps in treatment of cancer		
No	99	51.3
Yes	94	48.7
Plans to continue using CAM		
No	72	37.3
Yes	121	62.7

**Table 4:** CAM users' attitudes towards conventional health care.

Category	*N*	%
Disclosure of CAM use to healthcare provider		
Yes	74	38.3
No	119	61.7
Reason for nondisclosure		
Healthcare provider may dislike it	36	30.3
There is no need to disclose	76	63.9
I am not open-minded type	0	0
Healthcare provider does not know about CAM	1	0.8
Other	6	5.0
Any side effects of cancer therapy		
Yes	173	89.6
No	20	10.4

**Table 5:** Modalities of CAM used.

CAM	*N*	%
Dietary, nutritional therapy		
Mushrooms	90	46.6
Ginseng	103	53.4
Beans	84	43.5
Kale, Onion juice	54	28.0
Biological supplements		
Vitamins	78	40.4
Medicinal herbs (aloe, dandelion)	22	11.4
Supplements for blood circulation	17	8.8
Shark cartilage, Chitosan	10	5.2
Mind-body interventions		
Prayer, Meditation, Zen	66	34.2
Exercise, Yoga	104	53.9
Psychotherapy	14	7.3
Breathing	4	2.1
Manipulative and body-based therapies		
Massage	39	20.2
Sujichim (Korean hand acupuncture)	1	0.5
Acupressure	6	3.1
Chiropractic (Chuna therapy)	2	1.0
Alternative medicine		
Acupuncture	43	22.3
Traditional Korean Herbal Medicine	45	23.3
Moxibustion and Cupping	14	7.3
Physiotherapy	0	0
Other	3	1.6
